# Large landslides at the northeastern margin of the Bayan Har Block, Tibetan Plateau, China

**DOI:** 10.1098/rsos.180844

**Published:** 2019-01-16

**Authors:** Bo Zhao, Yunsheng Wang, Yonghong Luo, Ruifeng Liang, Jia Li, Lili Xie

**Affiliations:** 1 State Key Laboratory of Geohazard Prevention and Geoenvironment Protection, Chengdu University of Technology, Chengdu 610059, People's Republic of China; 2 Powerchina Kunming Engineering Corporation Limited, Kunming 650051, People's Republic of China

**Keywords:** large landslide, typical characteristics, control factors, northeastern margin of the Bayan Har Block

## Abstract

Large landslides (volume greater than or equal to 10^6^ m^3^) usually have disastrous consequences and clearly influence the evolution of the local landscape. In this study, a detailed investigation of large landslides, across 20 towns over an area of 5000 km^2^, was carried out on the northeastern margin of the Bayan Har Block, at the eastern margin of the Tibetan Plateau, China. The results show that there are 129 large landslides in this area. Among them, 79 landslides have volumes within 10^6^–10^7^ m^3^, 52 landslides have volumes within 10^7^–10^8^ m^3^ and 2 landslides have volumes larger than 10^8^ m^3^. Most of these landslides are distributed along rivers, and more than 32% are densely concentrated in three small regions. The landslides mainly occur in high slopes and exhibit obvious sturzstrom characteristics. Analysis of the factors controlling landslide occurrence shows that elevation, slope angle, slope aspect, lithology, faults and rivers (valley) clearly influence landslide occurrence, while rainfall has no obvious influence. Earthquakes are considered an important trigger of and contributor to landslide occurrence.

## 1. Introduction

The occurrence and frequency of landslides in an area fundamentally depend on the interaction between the triggering mechanisms and natural conditions, and a full understanding of their relationship is vital to assess natural hazards and to elucidate the role landsliding plays in driving landscape evolution in a range of settings [1–7]. Therefore, many famous landslide-prone regions have been adopted to study regional landslide occurrence laws [7–11]. For example, Eeckhaut *et al.* [12] revealed the frequency–area distribution derived from a detailed landslide inventory of the Flemish Ardennes (Belgium); Borgomeo *et al.* [13] used the Molise Mountain area in central Italy as a research object to study the spatial distribution and frequency of and geomorphic controls on landslide occurrence; additionally, Zhuang *et al.* [14] studied the distribution and characteristics of landslides in the Loess Plateau in Shaanxi Province. Migoń *et al.* [15] used a combination of LiDAR-derived digital elevation model (DEM) analysis and field mapping to reveal the influence of geomorphic diversity and the geological controls on landslide occurrence in the Kamienne Mountains, Central Europe.

**Figure 1.**
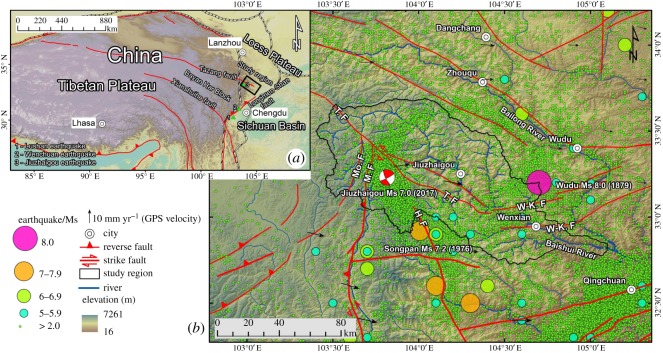
Regional setting and the location of the study region. M. F—Mingjiang fault, Mo. F—Mounigou fault, H. F—Huya Fault, W-K. F—Wenxian-Kangxian fault. The GPS velocities were downloaded from [16].

The eastern margin of the Tibetan Plateau, especially the Bayan Har Block, is a world-famous landslide-prone area (figure 1*a*) [17–20]. For example, the 2008 Wenchuan magnitude (Ms) 8.0 earthquake and the 2013 Lushan Ms 7.0 earthquake, which both occurred in the eastern margin of the Bayan Har Block (along the Longmen Shan fault) (figure 1*a*), have triggered more than 60 000 landslides and 4000 landslides, respectively [21,22]. Many researchers have begun to study the landslides in this area; for example, Ouimet *et al.* [23] studied the influence of large landslides on river incision in the northern margin of the Bayan Har Block. Xu *et al.* [24] revealed large landslide features triggered by the Wenchuan earthquake in the Longmen Shan region. Furthermore, another region requiring attention is the northeastern margin of the Bayan Har Block (figure 1*a*). This margin is also a region with concentrated geohazards: this region experienced a Ms 8.0 earthquake (the Wudu earthquake) in 1987 [25] and a Ms 7.0 earthquake (the Jiuzhaigou earthquake) in 2017 (figure 1*b*) [26]. To understand the landslide characteristics and their relationships with geomorphology and geology in the northeastern margin of the Bayan Har Block, a detailed field investigation and remote sensing interpretations were carried out to clarify the typical characteristics of large landslides (volume greater than 10^6^ m^3^); large landslides are the focus of this study because they can interrupt traffic, destroy buildings, block rivers and bury villages [27–31]. Then, the geomorphic and geologic control factors of large landslides are also discussed.

## 2. Regional setting and methodology

### 2.1. Regional setting

**Figure 2.**
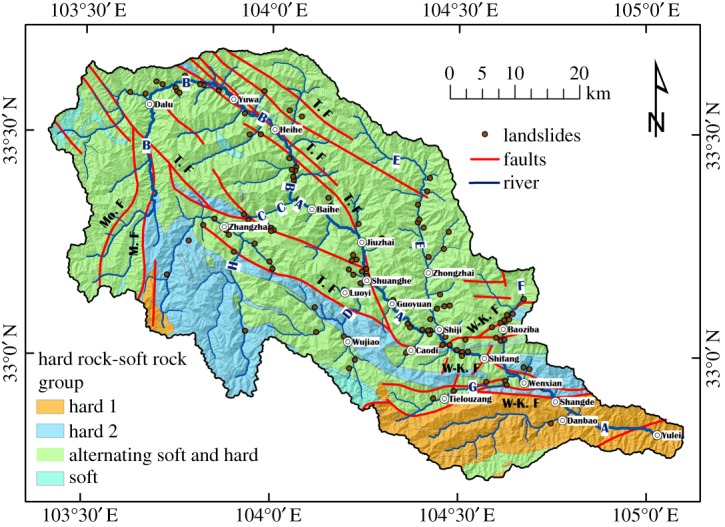
The rivers, town locations and rock group distribution in the study region. (A) Baishui River (mainstream), (B) Hei River, (C) Bai River, (D) Tangzhu River, (E) Zhonglu River, (F) Malian River, (G) Baimayu River; T. F—Tazang fault, M. F—Minjiang fault, Mo. F—Mounigou fault, W-K. F—Wenxian-Kangxian fault.

The study region is located in the transition zone between the Tibetan Plateau, Sichuan Basin and Chinese Loess Plateau and consists of 20 towns, covering a total area of 5000 km^2^, as shown in figure 1 and figure 2. The study region is also in the northeastern margin of the Bayan Har Block (figure 1*a*). The Bayan Har Block is a very active sub-block of the Tibetan block. For example, in the past 10 years, three very large earthquakes (the 2008 Wenchuan Ms 8.0 earthquake, 2013 Lushan Ms 7.0 earthquake and 2017 Jiuzhaiou Ms 7.0 earthquake) occurred along the eastern boundary (along the Longmen Shan fault range) and northern boundary (along the Tazang fault range) of the Bayan Har Block [26]. Figure 1*b* shows that the study region also includes a circular seismic belt.

The average GPS velocity (2009–2014) of the study region is approximately 6–10 mm yr^−1^ and exhibits primarily northeastward extrusion (figure 1*b*). The main faults present in the study region include the Tazang fault, Mounigou fault, Minjiang fault and Wenxian–Kangxian fault (figure 1*b*); the detailed fault distribution is shown in figure 2. Loess is concentrated locally in the study region, and the loess thickness mainly ranges from 8 to 30 m, with the maximum thickness reaching 50 m.

**Figure 3.**
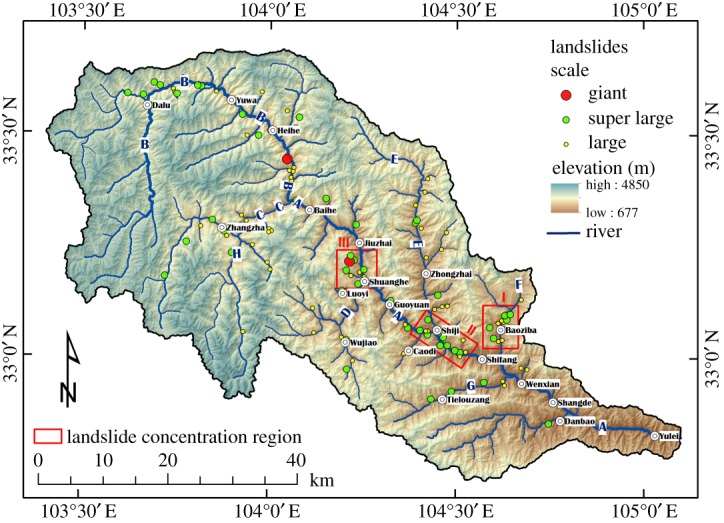
The distribution of large landslides. Large landslides—1–10 × 10^6^ m^3^, super large landslides—10–100 × 10^6^ m^3^, giant landslides—greater than 100 × 10^6^ m^3^; I—Baoziba region, II—Shiji region, III—Shuanghe region; (A)—Baishui River (mainstream), (B)—Hei River, (C)—Bai River, (D)—Tangzhu River, (E)—Zhonglu River, (F)—Malian River, (G)—Baimayu River.

The study region is characterized by densely distributed mountains and valleys. The gentle river valleys contrast with the steep mountainsides. The elevations in this area are high in the west (especially in the southwest, closer to the Tibetan Plateau) and low in the east, dropping from 3000–4800 m (western section) to below 1000 m (eastern section), as shown in figure 3. The elevation difference across the study region reaches 3500 m, and the highest elevation exceeds 4800 m.

The Baishui River flows through the study region (figure 1*b*). The Baishui River is a third-order tributary of the Yangtze River [32]. Influenced by faults in the study region, the Baishui River presents an approximately SE orientation (figure 2). There are seven main second-order tributaries from the Baishui River, as shown in figure 2. The average gradient of the Baishui River is approximately 10.1‰, its annual average runoff is 110 m^3^ s^−1^, and many deep and winding canyons are present on both sides of the river due to rapid erosion.

Owing to the large scale of the study region, the lithology varies and is divided into four different rock groups based on their properties: hard 1, hard 2, alternating hard and soft, and soft, as shown in figure 2. The detailed lithology of each rock group is listed in table 1. Figure 2 shows that most of the study region presents alternating hard and soft rock, which mainly consists of alternating limestone and slate and alternating metasandstone and phyllite (table 1). Alternating hard and soft rock is favourable for the occurrence of landslides. For example, recent catastrophic landslides of alternating metasandstone and phyllite (Xinmo landslide) occurred in Xinmo village, Maoxian County, Sichuan Province China, burying the entire Xinmo village and killing 84 people [33].

**Table 1.** The rock groups in the study region. Note: AnZbk—pre-Sinian Bikou group, P_2_d—middle Permian Dashibao group, Zbs—Sinian Shuijing group, D_3_xw—upper Devonian Xiawuna group, Cmh—Carboniferous Minhe group, P_1_s—lower Permian Sandaoqiao group, D_1_sh—lower Devonian Shifang group, D_2_d—middle Devonian Dangduo group, T_1_b—lower Triassic Bocigou group, T_2_zg—middle Triassic Zhagashan group, T_2_z—middle Triassic Zagunao group, T_3_zh—upper Triassic Zhuwo group, Zbw—Sinian Wugongkou group, T_3_xd—upper Triassic Xinduqiao group.

rock group	stratum	lithology
hard 1	AnZbk, P_2_d	amphibolite, migmatitic granite and basalt
hard 2	Zbs, D_3_xw, Cmh, P_1_s	limestone and dolomite
alternating hard and soft	D_1_sh, D_2_d, T_1_b, T_2_zg, T_2_z, T_3_zh	alternating limestone and slate, alternating metasandstone and phyllite
soft	Zbw, T_3_xd	phyllite and slate

At 21.19 local time (13.19 UTC) on 8 August 2017, a Ms 7.0 earthquake struck Jiuzhaigou County, Sichuan Province, China, with a focal depth of 20 km at 33.20° N, 103.82° E. This earthquake triggered more than 1780 landslides, damaged one dam (Nuorilang waterfall) and caused one dam to break (Huohua lake) [26]. Most of these landslides were small landslides (volume less than 10^4^ m^3^), and no large landslides were triggered. Although no large landslides occurred, many hills clearly exhibit fissures, decreasing their stability [26].

### 2.2. Method

In this research, a detailed field investigation and aerial photography were used to map the location, geomorphology and characteristics of large landslides.

A detailed field investigation was conducted using a hand-held global positioning system (GPS), laser rangefinders and field records. The material composition, boundaries and other parameters of the landslide deposits were determined and recorded. The slope structure and lithology of the source area, as well as evidence of the failure mechanism and evolution of the landslides, were also collected. Aerial photography, with a spatial resolution of 0.53–1.03 m, was used to map the location and boundaries of the landslides.

After completing the field investigation, we used ArcGIS software to overlay all the data with topographic and geologic information to assess the distribution patterns. Based on these survey results, a comprehensive analysis was adopted to study the typical characteristics of the large landslides.

## 3. Typical characteristics of large landslides

### 3.1. Basic characteristics

The field investigation suggests that there are 129 large landslides concentrated in the study region, as shown in figure 3. Based on the classification criteria for large landslides [24], they can be divided into large landslides (volume: 1–10 × 10^6^ m^3^), super large landslides (volume: 10–100 × 10^6^ m^3^) and giant landslides (volume greater than 100 × 10^6^ m^3^), and the number of each of these types of landslides in this area are 75, 52 and 2, respectively. We use ‘large’ to describe both large, super large and giant landslides, that is, those with debris volumes greater than 10^6^ m^3^. Based on the landslide deposit composition, the landslides in the study region can be divided into rock landslides and mixed soil-rock (loess-rock) landslides. The field investigation shows that 104 of the landslides are rock landslides, while the other 25 landslides are mixed loess-rock landslides.

**Figure 4.**
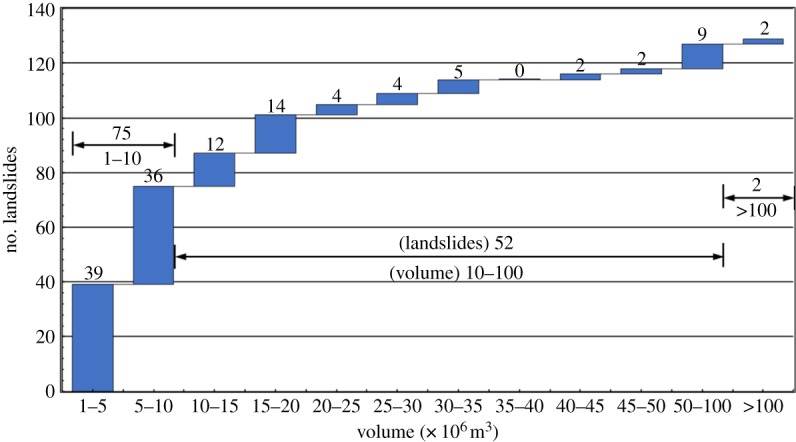
Scale concentration laws of the large landslides in the study region.

Figure 4 shows the detailed landslide scale and distribution patterns. The average landslide volume is 17.23 × 10^6^ m^3^, and as the landslide volume increases, the number of landslides decreases rapidly (figure 4). In the study region, 75 landslides have volumes of 1–10 × 10^6^ m^3^, accounting for 58.1% of the total landslides; 78.3% of the landslides have volumes of less than 20 × 10^6^ m^3^; 8.5% of landslides have volumes larger than 50 × 10^6^ m^3^ and 2 landslides have volumes greater than 100 × 10^6^ m^3^. The largest landslide in the study region is the Gaoerfu#1 landslide (104.2158° E, 33.2157° N), which has a volume of approximately 105 × 10^6^ m^3^.

### 3.2. Distribution characteristics

#### 3.2.1. Distribution laws along rivers

Based on the field investigation, most of the landslides concentrate along rivers (figure 3). Table 2 shows the distribution of large landslides along different rivers.

**Table 2.** The distribution of large landslides along different rivers.

river	class	number of landslides	%
Baishui River	mainstream	25	19.4
Hei River	tributary	26	20.2
Bai River		19	14.7
Tangzhu River		8	6.2
Zhonglu River		14	10.9
Malian River		13	10.1
Baimayu River		5	3.8
other rivers		19	14.7

Table 1 and figure 3 show that most of the large landslides occur along rivers, with 91 landslides concentrated along the mainstream of the Baishui River and the Hei River, Bai River, Malian River and Zhonglu River tributaries, accounting for 70.5% of the total landslides.

In particular, 25 landslides are located along the mainstream of the Baishui River and account for 19.4% of the total landslides; 104 landslides are located along the tributaries of the Baishui River and account for 80.6% of total landslides. Along the tributaries, the Hei River hosts 26 landslides (20.2%), which is more than the mainstream of the Baishui River (table 2). Therefore, the majority of the recorded landslides (80.6%) concentrate along the tributaries of the Baishui River.

#### 3.2.2. Uniformity of distribution

The field investigation suggests that the distribution of large landslides is clearly not uniform. Most landslides are concentrated in the middle reaches of the mainstream of the Baishui River, and in the middle and lower reaches of tributaries, while few landslides occurred in the lower reaches of the mainstream of the Baishui River and its source rivers (figure 3).

**Figure 5.**
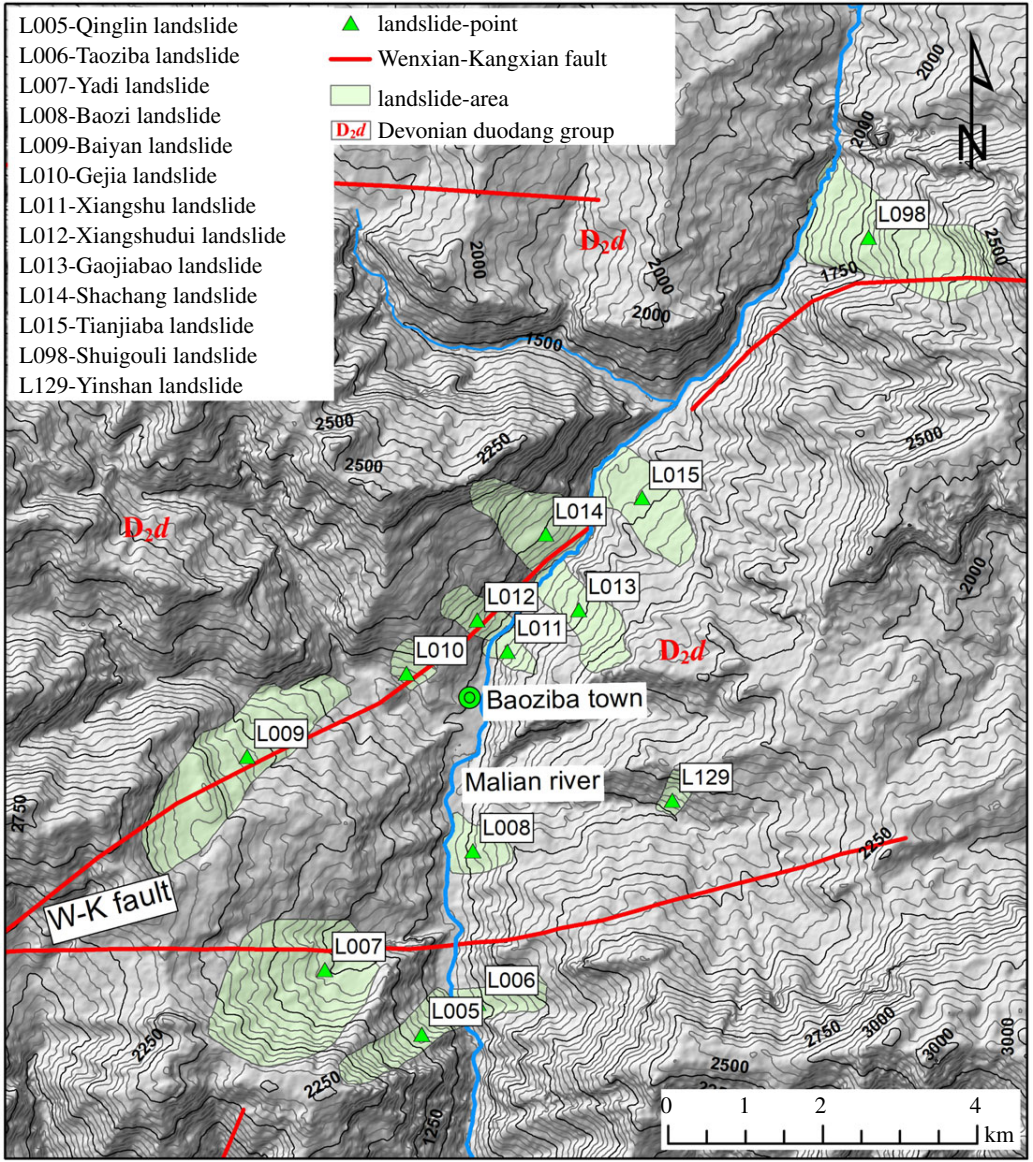
The large landslides concentrated in the Baoziba region.

**Figure 6.**
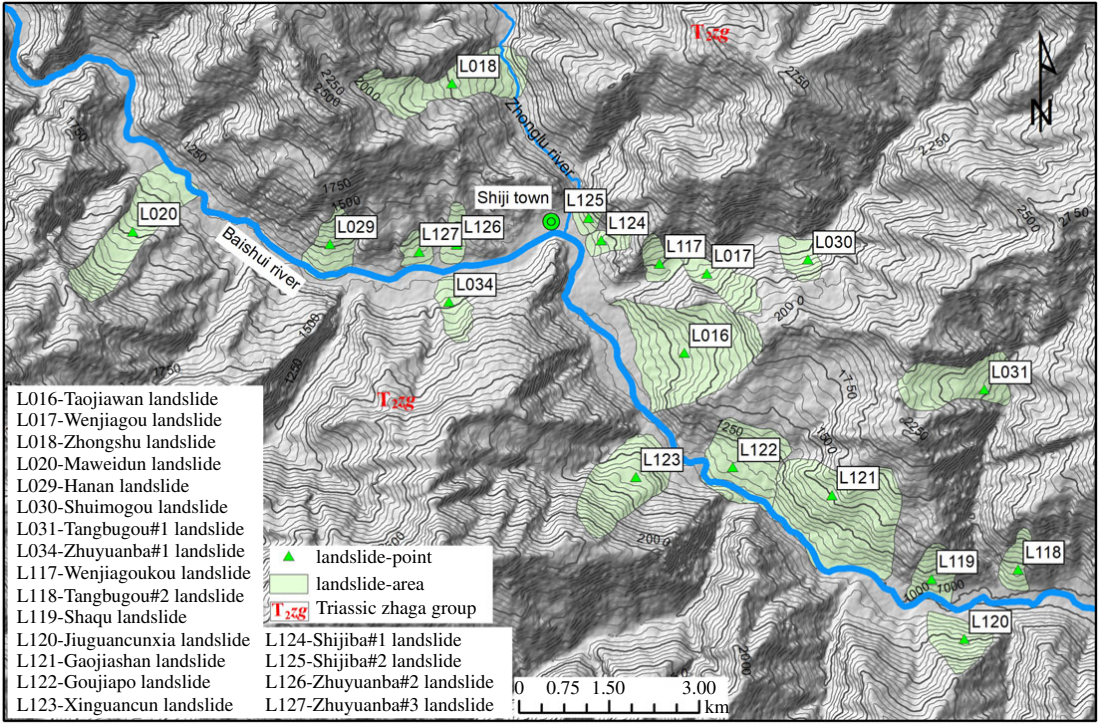
The large landslides concentrated in the Shiji region.

**Figure 7.**
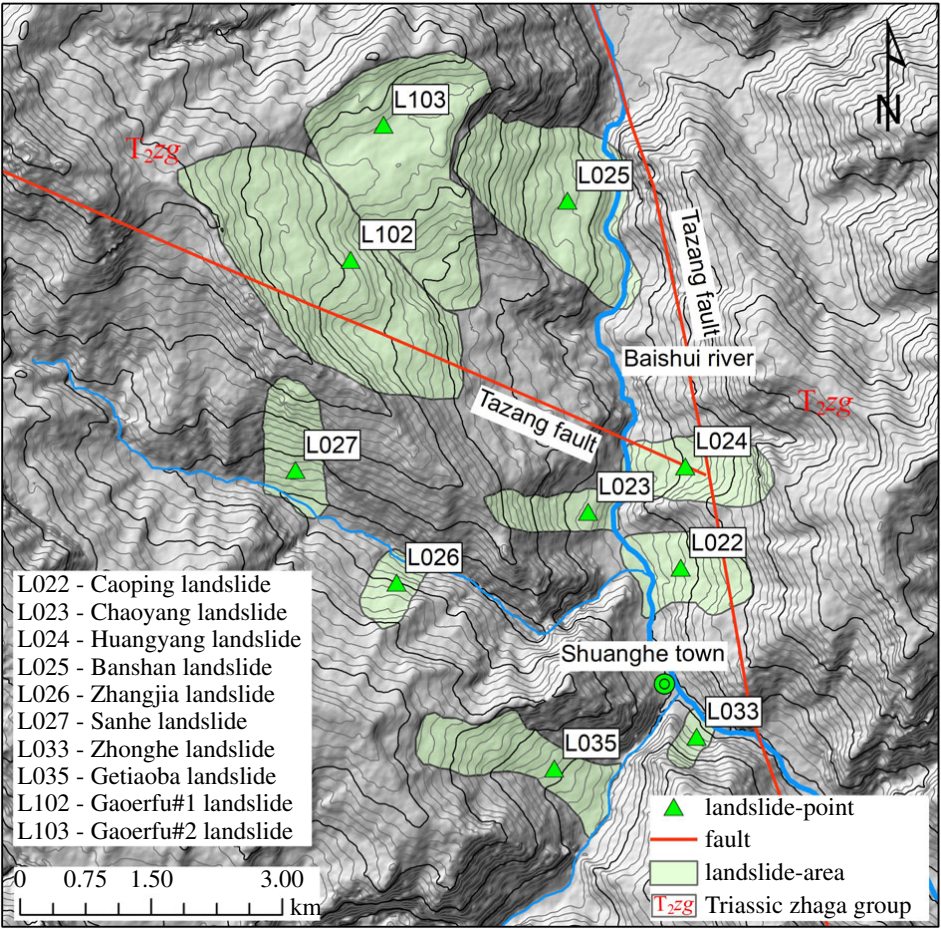
The large landslides concentrated in the Shuanghe region.

There are three main regions with a concentrated distribution of large landslides (figure 3): the Baoziba region of the Malian River (figure 5) and the Shiji and Shuanghe regions of the Baishui River (figures 6 and 7). In these three regions 42 landslides occur, accounting for 32.6% of the total landslides.

##### 3.2.2.1. Baoziba region

There are 13 landslides concentrated in the Baoziba region (figure 5), reflecting a linear density of up to 0.97 km^−1^. The strong river incision in this region has created very high and steep slopes. The alternating phyllite and limestone rock group (D_2_*d*) is widely distributed, and this rock group provides the basic conditions for the occurrence of landslides. The Wenxian–Kangxian fault crosses this region (figure 5), affecting the occurrence of landslides in this region. The slope structure of the river bank in this region is mainly a transverse slope.

##### 3.2.2.2. Shiji region

There are 19 landslides concentrated in the Shiji region (figure 6), reflecting a linear density of up to 1.12 km^−1^. The river valleys in this region are mainly V-type valleys. The alternating limestone and slate rock group (T_2_*zg*) is widely distributed, and this alternating soft and hard rock group causes landslide occurrence easily. Owing to the V-type valley, the slope structures in this region are mainly moderate-steep bedded slopes (left bank) and moderate-steep reverse slopes (right bank). In addition, most landslides occurred in the bedded slopes (left bank).

##### 3.2.2.3. Shuanghe region

There are 10 landslides concentrated in the Shuanghe region (figure 7), reflecting a linear density of up to 0.95 km^−1^. The river valleys in this region are mainly V-type valleys. The rock groups in this region are mainly alternating limestone and slate (T_2_*zg*) and alternating metasandstone and phyllite (T_2_*zh*), which are both alternating soft and hard rock groups. The Tazang fault crosses this region (figure 7). The Tazang fault is a very active fault; the 2017 Jiuzaigou Ms 7.0 earthquake occurred along the Tazang fault [26].

### 3.3. Movement characteristics

**Figure 8.**
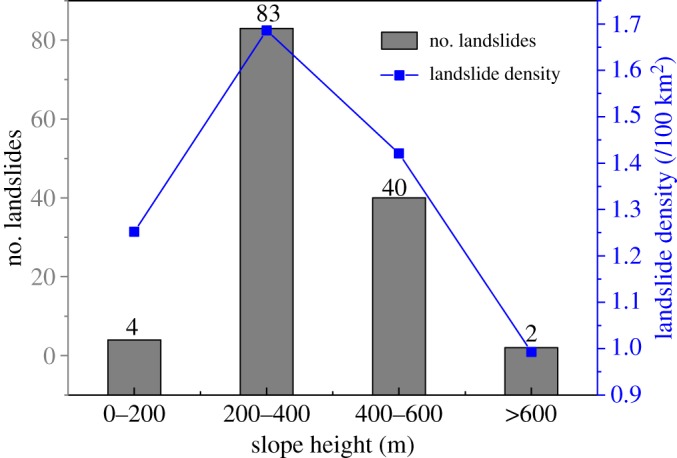
The relationship between slope height and number of landslides.

Figure 8 shows the relationship between the number of landslides and slope height. Figure 8 shows that the slope heights of 125 landslides exceed 200 m, accounting for 96.8% of the total landslides. More than 95% of the landslides were located at heights of 200–600 m. These results may indicate that many large landslides concentrate in the high areas of slopes. Based on our previous studies, high slopes usually induce more obvious site amplification than that at lower positions under seismic conditions [26,33].

To evaluate the motion characteristics of these large landslides, this study uses the equivalent friction coefficient *f* proposed by Shreve [34], which is defined as follows:

3.1 $$f=\frac{H}{L},$$

where *f* is the equivalent friction coefficient, *H* is the height of the highest point on the breakaway rim and *L* is the travel distance of the slide mass. The *f* value can reflect the landslide mobility: the smaller the value, the higher the mobility [28].

**Figure 9.**
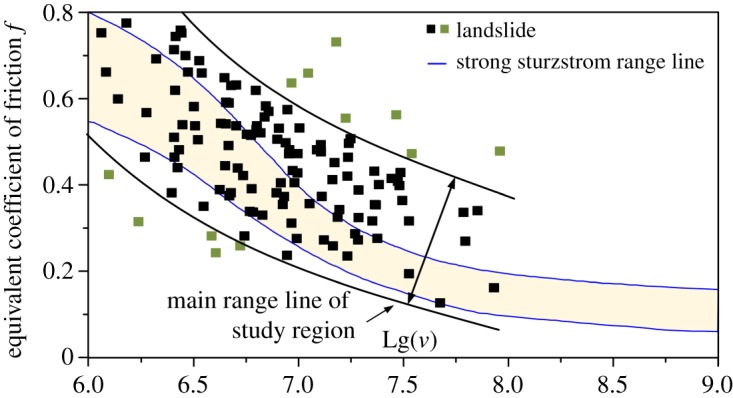
Movement characteristics of the landslides. Lg(*v*) = Log_10_(*v*), where *v* is landslide volume (m^3^).

The *f* values of the large landslides have been determined and are shown in figure 9. Figure 9 shows that as the landslide volume increases, *f* generally decreases. This relationship may indicate that larger landslides can travel longer distances.

Hsü obtained the relationship between the *f* value and landslide volume for sturzstrom-type landslides (see the strong sturzstrom range lines in figure 9) [35]. As shown in figure 9, more than 40% of the large landslides present motion characteristics typical of sturzstrom; these landslides are all rock landslides and generally have the characteristics of a high slide position and adequate motion space. Furthermore, according to the field investigation, the elevation difference between the source zone and the accumulation zone is generally more than 350 m, the slope is greater than 30°, and few large barriers exist in the slide channels.

### 3.4. Analysis of a typical landslide

This section discusses the characteristics and mechanisms of a typical landslide (the Taojiawan landslide).

#### 3.4.1. Basic description

**Figure 10.**
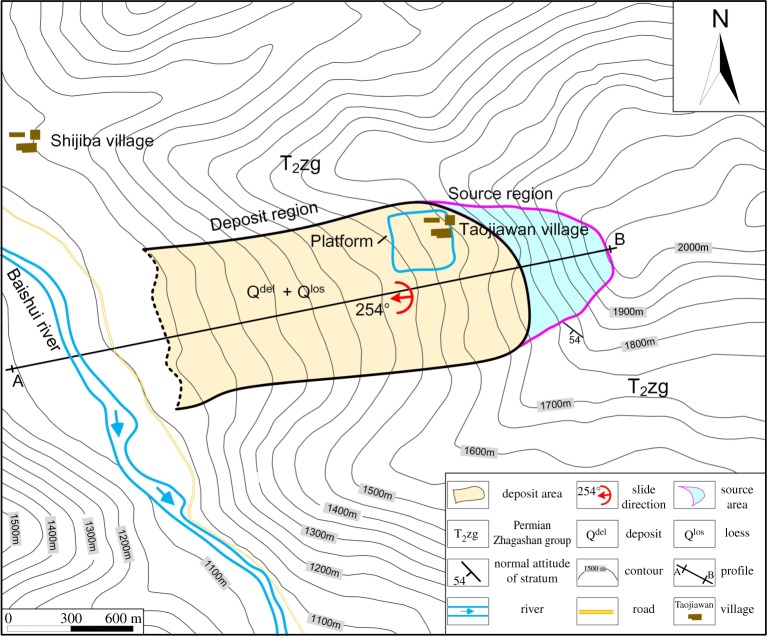
Overview of the Taojiawan landslide.

The Taojiawan landslide is located along the mainstream Baishui River (104.4665° E, 33.0458° N). The entire landslide follows a WSW direction and is approximately perpendicular to the river trend (figure 10). Various gullies, induced by rainfall, cut the landslide deposit into different discontinuous segments. Based on the field investigation, the elevation range of the Taojiawan landslide is approximately 85 m, the length is 1700 m, the width is 830 m, the mean thickness is approximately 60 m and the volume is approximately 71.4 × 10^6^ m^3^. Therefore, the Taojiawan landslide is a super large landslide.

#### 3.4.2. Typical characteristics

**Figure 11.**
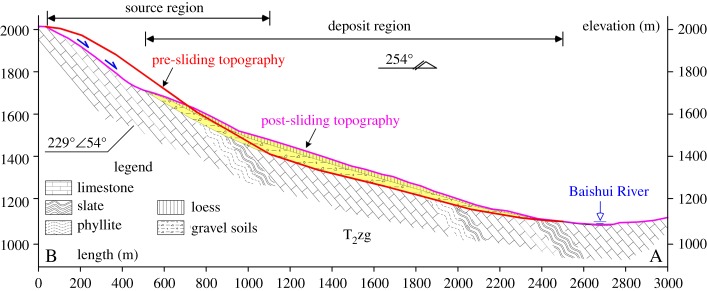
The section profile of the Taojiawan landslide (AB line in figure 10).

**Figure 12.**
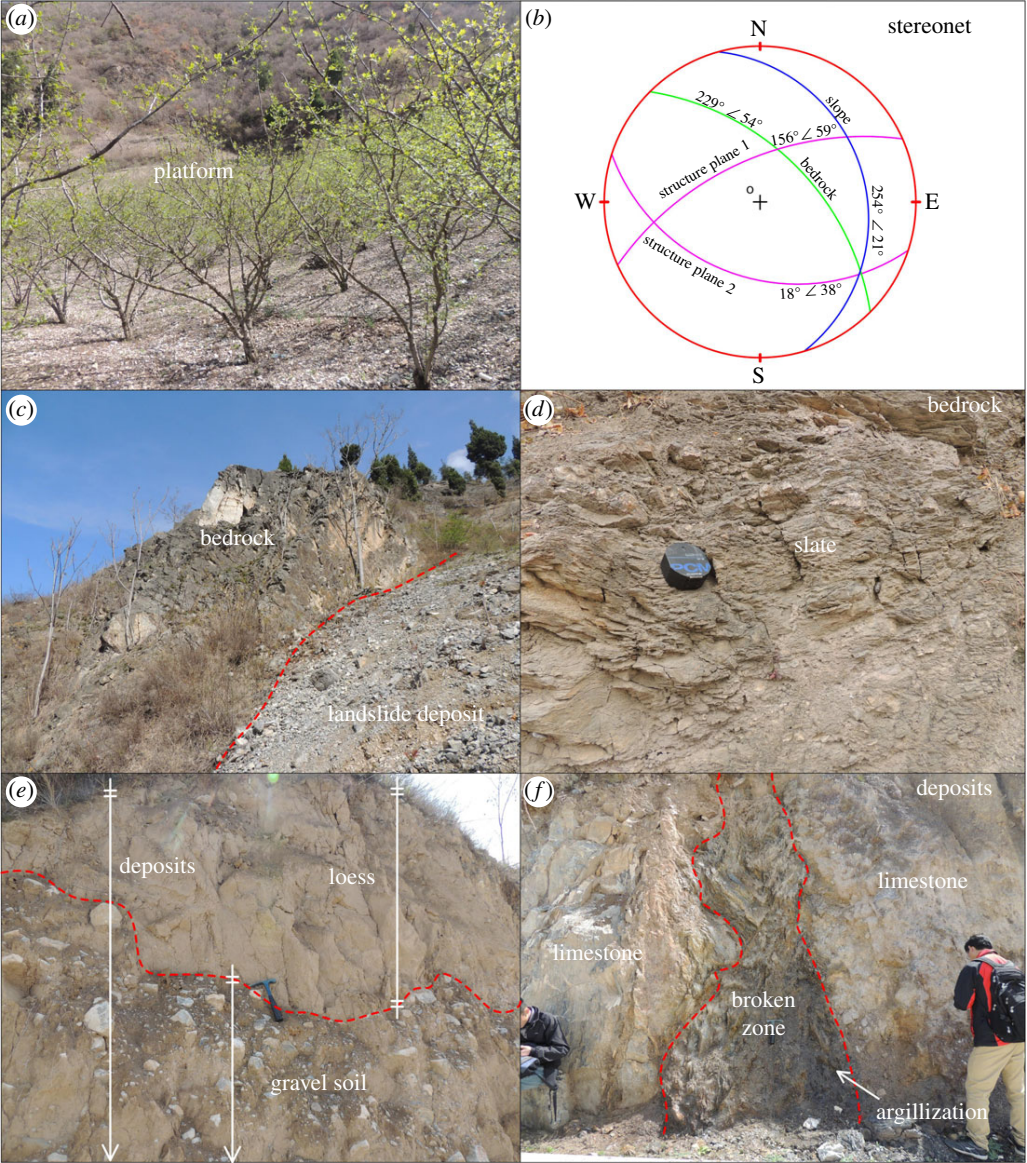
Local characteristics and stereonet of the Taojiawan landslide. (*a*) The platform of the Taojiawan landslide (location shown in figure 10), (*b*) the stereonet of the Taojiawan landslide, (*c*) bedrock exposed at rear scarp, (*d*) slate exposed at slope foot, (*e*) landslide deposit, (*f*) broken zone.

A section profile (AB in figure 10) is shown in figure 11. At the head scarp of the landslide, the exposed bedrock (figure 12*c*) is limestone of the middle Permian Zhagashan group (T_2_*zg*) with medium weathering, whose orientation is 229°∠54° (figure 11). Two structure planes can be observed in the bedrock: I, 156°∠59°; and II, 18°∠38°, whose stereographic projection is shown in figure 12*b*.

Under the source region, there is a platform with a width of 400 m and a length of 300 m (figures 10 and 12*a*). Taojiawan village is located on this platform. The elevation of the toe section of the landslide is approximately 1100 m, with a partially covered I terrace and no obvious shear outlets. Slate with obvious deformation is observed in the slope foot (figure 12*d*). The landslide deposits are mainly composed of loess soil and gravel soil, the loess soil usually covered the gravel soil (figure 12*e*). The maximum thickness of the exposed loess deposit is approximately 12 m. Owing to the construction of roads, fake bedrock from within the landslide deposits is exposed in front of the landslide deposits (figure 12*f*); the broken zone within this fake bedrock is composed of broken phyllite and is undergoing local argillization. In addition, the outcrops of limestone on either side of the broken zone are clearly different.

#### 3.4.3. Mechanism of the Taojiawan landslide

The Taojiawan landslide is located on the left bank of the Baishui River. Owing to deep river incision, the slope body has enough space for deformation, and the slope foot is eroded. The rock groups are alternating soft and hard rock, and the soft rock (slate) is weak in terms of the resistance to weathering and deformation. Based on its slide direction and rock type distribution, the Taojiawan landslide is a bedding landslide. In addition, the landslide is located in an area with an earthquake intensity of VIII, the rock masses are fractured and two structure planes are present, the stereographic projection of which is shown in figure 12*b*.

Based on the field investigation, the mechanism of the Taojiawan landslide can be summarized as follows:

(1) With the regional crustal uplift and rapid incision of the Baishui River, the stress in the rock mass was redistributed; the shear stress gradually concentrated at the slope foot, and numerous unloading fissures and tension fissures formed in the rock mass. The rapid incision made the slope foot a free surface, and the soft slate began to deform under the action of landslide thrusting, water, earthquakes, etc.
(2) With time, the soft slate began to deform faster, and the slope body also began to deform. This caused the rock mass to be potentially unstable.
(3) With the accumulation of deformation, the fractures in the slope foot increased, and its resistance to deformation decreased rapidly. Finally, some trigger factors, such as earthquakes and rainfall, triggered the landslide.

## 4. Geomorphic and geologic controls on large landslides

### 4.1. Control by topographic and geomorphic factors

The topographic and geomorphic factors discussed in this section mainly include elevation, slope angle and slope aspect.

#### 4.1.1. Elevation

**Figure 13.**
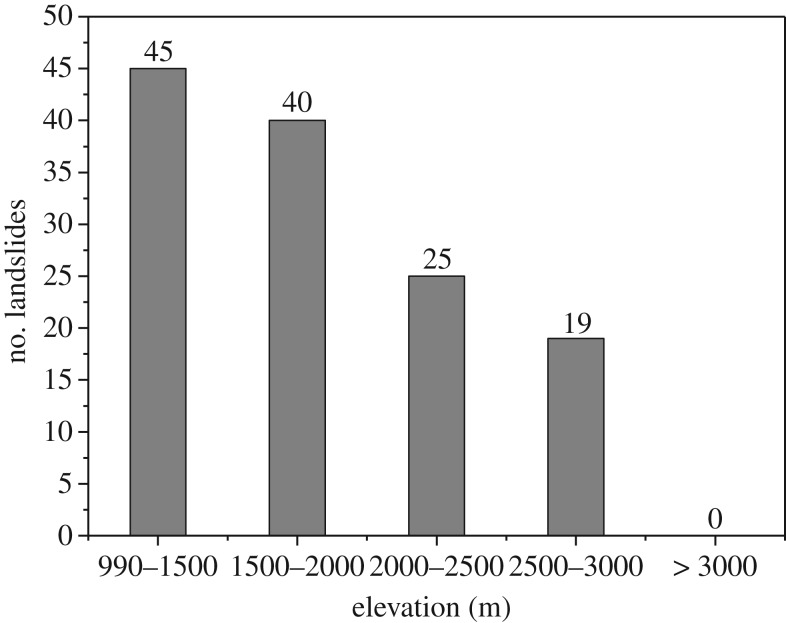
The relationship between elevation and number of landslides.

As illustrated in figure 13, all the landslides occurred within an elevation range of 1000–3000 m; 68% of the large landslides occurred within an elevation range of 990–2000 m. At higher elevations, the landslide occurrence clearly decreases. In addition, more landslides occur at elevations of 990–1500 m than at other elevation ranges.

Based on the field investigation, the elevation range of 1000–2600 m (upstream: 2200–2600 m, midstream: 1600–2200 m, downstream: 1000–1600 m) coincides with the position where the valley transitions from a broad valley to a gorge, and most of the landslides occurred in the upper part of the gorge. Similar conclusions were also observed following the Wenchuan earthquake [36].

#### 4.1.2. Slope angle

**Figure 14.**
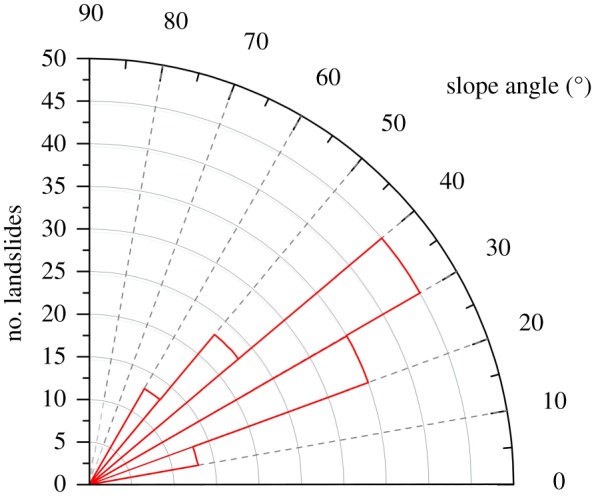
The relationship between landslide and slope angle.

Slope angle has a significant influence on landslide occurrence. To analyse the influences of slope angle on landslide distribution, the slope angle was calculated from the data collected in the field investigation. Then, a slope map was constructed with intervals of 10° (figure 14).

Figure 14 illustrates that the number of landslides increases with the slope angle, exhibits a maximum for slopes of 30–40° and then greatly decreases for slopes of 40–50°. Slope angles of 20–40° correspond to more than 62% of the landslides. Our field investigation shows that very steep slopes (greater than 50°) rarely develop large landslides because they are already in an unstable state, causing rockfalls or small landslides to occur frequently; thus, not enough source materials are available for the occurrence of large landslides. Similar results were reported by Parise & Jibson [37], Wasowski *et al.* [38] and Qi *et al*. [39] for co-seismic landslides in different parts of the world.

#### 4.1.3. Slope aspect

**Figure 15.**
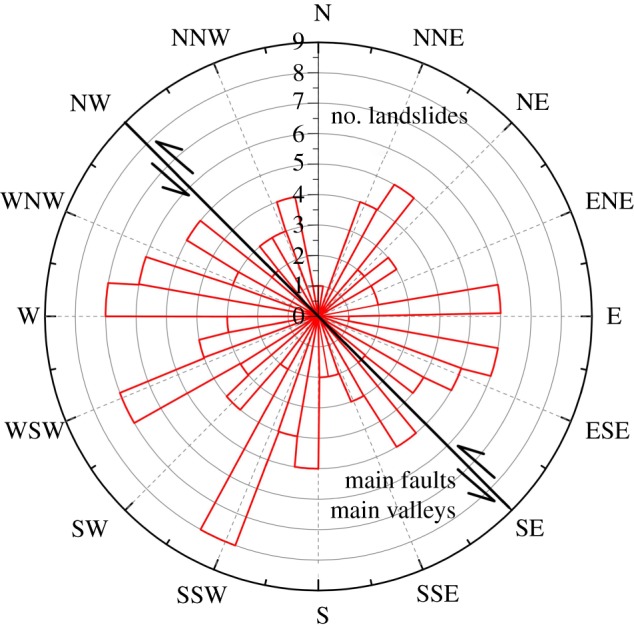
Distribution of slide direction.

Unlike the random slide directions of small and medium landslides, the slide directions of the large landslides in this area have a clear regularity. As shown in figure 15, the slide directions of most landslides are to the SSW–WNW and ENE–SSE, accounting for more than 65% of the total landslides. Only a few landslides occurred in other directions.

From a regional perspective, the majority of the mainstream of the Baishui River and many of its tributaries exhibit a SE orientation, and the majority of landslides occurred along rivers that follow this SE direction. The incised valleys provide enough free surfaces (oriented in the SW and NE directions) for landslide occurrence. The unloading of rock mass and river erosion make the slopes along the valleys potentially unstable. Frequent earthquakes or other factors, such as rainfall, in this region ultimately trigger these landslides.

### 4.2. Control by lithology and faults

#### 4.2.1. Lithology

**Figure 16.**
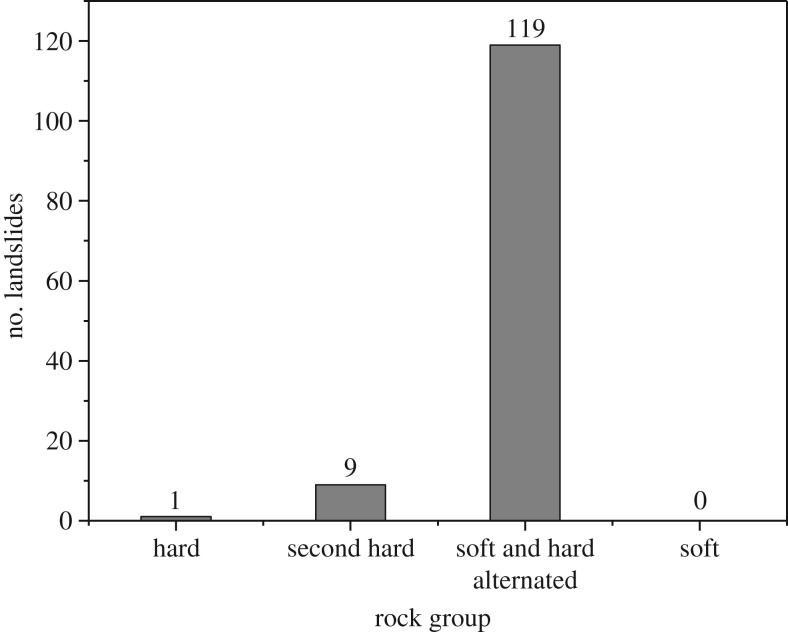
The landslide distribution in different rock groups.

Figure 16 shows the distribution of landslides within the four rock groups discussed in the previous section.

As illustrated in figure 16, 119 landslides occurred in the alternating soft and hard rock group, accounting for more than 92% of the total landslides. Alternating soft and hard rock, such as limestone and slate or metasandstone and phyllite, have poor properties: the cohesive strength between the hard rock and weak rock is very low, and the mechanical properties of the soft rock are also very poor. The soft rock can be considered weak interlayers within the hard rock, potentially cutting the hard rock into discontinuous segments and more easily inducing landslides compared to the other rock groups.

#### 4.2.2. Faults

**Figure 17.**
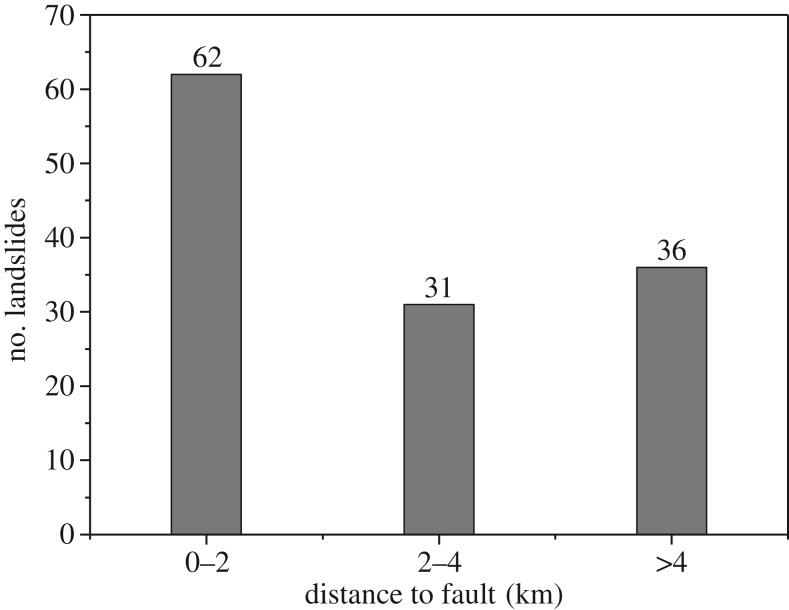
Spatial relationship between the landslides and faults.

The study region is located in a seismic belt (figure 1), and analysis of the spatial relationship between the faults and landslides is necessary. Figure 17 shows the variations in the landslide distribution with distance from the faults.

Figure 17 shows that the number of landslides decreases with distance to a fault, with the highest landslide concentration occurring at a distance of 2 km, which accounted for approximately 48% of the landslides, and more than 72% of the landslides occurred within 4 km of a fault. Therefore, faults have an obvious influence on the occurrence of large landslides.

In addition, the faults in the study region are very active, the small earthquakes (Ms less than 3.5) occurred frequently, according to reports from the China Earthquake Administration [40]. Additionally, an Ms 7.0 earthquake occurred at Zhangzha town, Jiuzhaigou County on 8 August 2017 [26]. Therefore, special attention should be paid to active faults.

### 4.3. Control by hydrography

#### 4.3.1. River and valley

**Figure 18.**
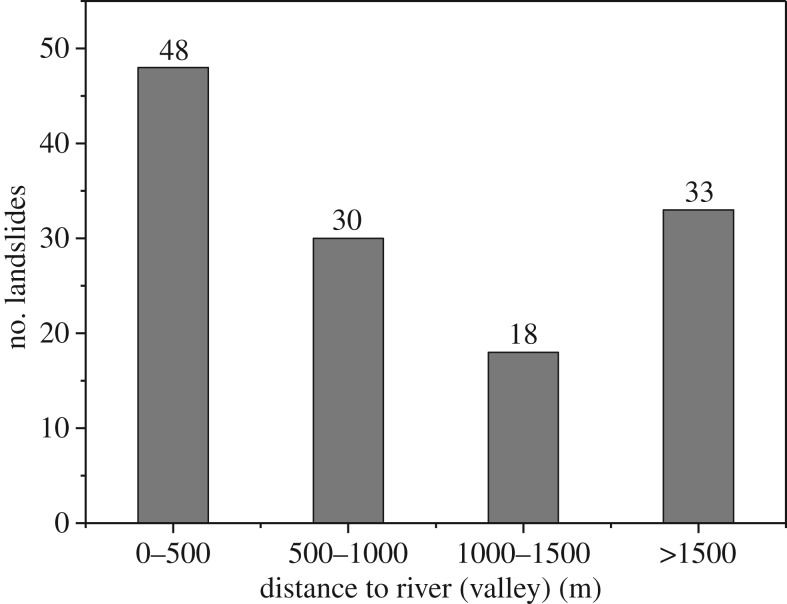
Spatial relationship between the landslides and rivers (valleys).

Rapid river incision provides enough space for the deformation of landslides and cuts the slope foot directly. Therefore, rivers are an important control factor of these landslides. Because rivers follow valley bottoms, the spatial relationships between the landslides and the rivers and valleys can be calculated simultaneously. Figure 18 shows the spatial relationship between the landslides and rivers (valley).

Figure 18 shows that the number of landslides decreases as the distance to the river increases; 48 landslides occurred within 0.5 km of a river, accounting for 37% of the total landslides, and more than 75% of the landslides occurred within 1.5 km of a river.

In addition, figure 18 also shows that most of the large landslides occurred in valleys. The valley areas exhibit a high concentration of landslides because they provide advantageous topographic, slope and dynamic conditions.

#### 4.3.2. Rainfall

**Figure 19.**
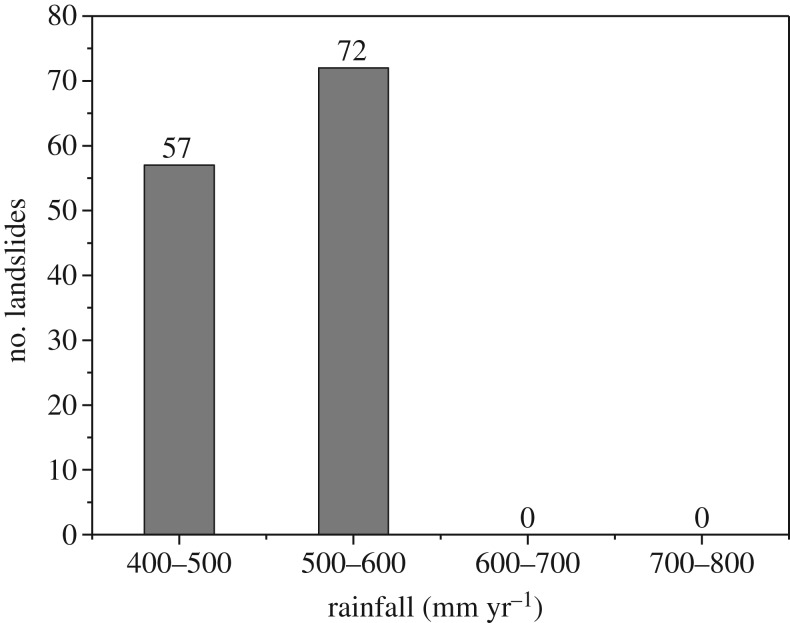
Relationship between landslide and rainfall.

Based on the distribution of the annual average rainfall in the study region, the numbers of large landslides in different annual average rainfall classes are calculated, as shown in figure 19.

Figure 19 shows that with an increase in rainfall, the number of landslides does not increase. For example, in areas with high rainfall (600–700 and 700–800 mm yr^−1^), no large landslides were observed, while in areas with low rainfall, the landslides are densely concentrated. Therefore, there is no obvious relationship between rainfall and landslide distribution.

## 5. Discussion

Based on the field investigation and comprehensive analysis, earthquakes may be an important trigger factor or contributor to landslide occurrence; the main reasons are as follows.

The study region has a seismic intensity of VIII and experiences peak accelerations of 0.2–0.35*g* [41]. Many earthquakes have occurred in this region, with the exception of small earthquakes (2.0 > Ms > 5.0) (figure 1*b*). Many large earthquakes, such as the Wudu Ms 8.0 earthquake, Songpan Ms 7.2 earthquake and Jiuzhaigou Ms 7.0 earthquake, also have occurred in this region. In addition, as illustrated in figure 1, a circular seismic belt clearly crosses the study region; most earthquakes concentrate within this seismic belt. This pattern of a central seismic gap has been noted as premonitory phenomena useful for earthquake prediction [42–46]. These results indicate that the faults in the study region are very active, and strong dynamic conditions can trigger large landslides or continuously weaken their stability.

In addition, in addition to earthquakes, rainfall, glacial activity and climate change are also contributors to and possible trigger factors of landslides.

Compared with other regions in the eastern margin of the Tibetan Plateau, the large landslides in the study region have the following characteristics:

(1) Zhao *et al.* [47] and Wang *et al.* [48] indicated that large landslides in the Dadu River and Longmen Shan areas are distributed along rivers, mainly concentrated along the mainstreams. The large landslides in the study region are also distributed along rivers, while more than 80% of the landslides occur along tributaries instead of the mainstream.
(2) In the study region, three areas with clear landslide clustering account for 32.6% of the total landslides. This is an uncommon phenomenon in other regions, with the exception of the Diexi region along the Minjiang River [33]. Further investigations could be carried out to identify the causes of this type of spatial distribution.
(3) Earthquakes are considered both an important trigger factor of and a contributor to landslide occurrence in this study region and other regions [14,18,19,22]. A circular seismic belt with a central gap occurs within the study region, and no similar phenomena appear in other regions.
(4) Similar to this study region, in other regions, most rock landslides occur on high slopes and present sturzstrom movement features [49]. Additionally, faults and rivers have an obvious influence on the landslide distribution [21,22]. Other factors, such as rainfall, lithology and elevation, influence landslides differently in different regions of the eastern margin of the Tibetan Plateau.

## 6. Conclusion

To clarify the characteristics of large landslides in the northeastern margin of the Bayan Har Block, a detailed field investigation was carried out. Based on a field investigation and comprehensive analysis, some conclusions can be drawn as follows:

(1) There are 129 landslides concentrated in the study region, and 75 landslides have volumes of 1–10 × 10^6^ m^3^, accounting for 58.1% of the total landslides; 78.3% of the landslides have volumes less than 20 × 10^6^ m^3^; 8.5% of the landslides have volumes greater than 50 × 10^6^ m^3^; and 2 landslides have volumes greater than 100 × 10^6^ m^3^. Regarding the deposit composition, the landslides can be divided into rock landslides (104 landslides) and mixed loess-rock landslides (25 landslides).
(2) Most large landslides occurred along rivers, specifically in the Baoziba region of the Malian River and the Shiji and Shuanghe regions of the Baishui River; 42 landslides were observed within these three regions, accounting for 32.6% of the total landslides.
(3) A total of 96.8% of the large landslides occurred on high slopes (slope height greater than 200 m), and approximately 40% of large landslides present motion characteristics typical of sturzstrom.
(4) Control factors, such as elevation, slope angle, slope aspect, lithology, faults and rivers (valleys), influence landslide occurrence. However, rainfall has no obvious influence on landslide occurrence.
(5) Earthquakes are considered an important trigger factor of and contributor to landslide occurrence.

## Supplementary Material

Detailed landslide information

## Data accessibility

The information of landslides can be found in the electronic supplementary material; other data can be found within the manuscript.
