# Supplementary material for: Large landslides at the northeastern margin of the Bayan Har Block, Tibetan Plateau, China
Source: R Soc Open Sci. 2019 Jan 16;6(1):180844. doi: 10.1098/rsos.180844 (PMC6366217; doi:10.1098/rsos.180844)
Supplement: Detailed landslide information [file rsos180844supp1.pdf]

Journal: Royal Society Open Science

Title: Large Landslides at the Northeastern Margin of the Bayan Har Block, Tibetan Plateau, China

Detailed landslide information (Supplementary materials)

Bo Zhao[1], Yunsheng Wang[1], Yonghong Luo[1], Ruifeng Liang[2], Jia Li[1], Lili Xie[1]

1. State Key Laboratory of Geohazard Prevention and Geoenvironment Protection, Chengdu University of Technology, China 610059;

2. Powerchina Kunming Engineering Corporation Limited, Kunming 650051, China.

Number	Landslides	E (°)	N (°)	Elevation (m)		Slide direction (°)	Volume (10^4m^3)	Scale
				Toe	Head scarp			
HP001	Xujiaba	104.7219	32.9346	927	1450	200	840	Large
HP002	Guajiagou	104.6903	32.9746	1357	1950	275	660	Large
HP003	Lijiagou	104.6746	32.9786	1426	2013	125	936	Large
HP004	Jintiaoshan	104.6256	32.9484	1028	1396	142	900	Large
HP005	Qinglinpo	104.6140	33.0379	1160	1880	60	690	Large
HP006	Taojiaba	104.6234	33.0402	1170	1510	258	785.4	Large
HP007	Yadixia	104.6010	33.0444	1430	1750	75	1922.8	Super large
HP008	Baoziba	104.6211	33.0578	1225	1434	275	840	Large
HP009	Baiyanshan	104.5901	33.0684	1530	2250	45	6240	Super large
HP010	Gejiabadui	104.6126	33.0783	1380	1500	230	384.8	Large
HP011	Xianshuwan	104.6265	33.0808	1280	1500	310	421.2	Large
HP012	Xiangshuwandui	104.6221	33.0843	1300	1600	120	566.7	Large
HP013	Goajiabao	104.6357	33.0858	1310	1740	316	1566	Super large
HP014	Shachang	104.6303	33.0955	1340	1840	118	1710	Super large
HP015	Tianjiabao	104.6452	33.0983	1370	1710	320	3360	Super large
HP016	Taojiawan	104.4665	33.0458	1110	1720	258	7140	Super large
HP017	Wenjiagou	104.4705	33.0574	1280	1510	323	673.2	Large
HP018	Zhongshuping	104.4259	33.0855	1180	1900	80	1932	Super large
HP019	Qiangouping	104.6299	32.9388	1240	1515	308	822.2	Large
HP020	Maweidun	104.3721	33.0672	1180	1850	35	2250	Super large
HP021	Goulicun	104.3035	33.1661	1670	2060	104	504	Large
HP022	Xiangping	104.2555	33.1814	1320	1590	275	990	Large
HP023	Chaoyangcun	104.2431	33.1913	1440	1600	102	607.5	Large
HP024	Huangyangcun	104.2543	33.1974	1340	1780	270	2312.8	Super large
HP025	Banshancun	104.2283	33.2182	1510	2400	116	9540	Super large
HP026	Zhangjialiang	104.2201	33.1840	1630	1950	20	868	Large
HP027	Sanhecun	104.2074	33.1955	1780	2200	185	1111	Super large
HP028	Luoyixiang	104.1955	33.1502	1670	2050	187	1710	Super large
HP029	Hanan	104.4045	33.0617	1140	1760	175	1770	Super large
HP030	Shuimogou	104.4623	33.0590	1245	1400	130	352.8	Large
HP031	Tangpubo#1	104.5196	33.0404	1680	2010	85	924	Large

HP032	Guoyuanxiang	104.3266	33.1280	1230	1670	240	1536	Super large
HP033	Zonghecun	104.2572	33.1679	1285	1540	28	257.6	Large
HP034	Zhuyuanba#1	104.4244	33.0519	1160	1460	2	1128	Super large
HP035	Getiaobacun	104.2391	33.1651	1330	1960	118	1934.4	Super large
HP036	Shawucun	103.6602	33.5854	2600	3240	191	2970	Super large
HP037	Congyacun	103.9250	33.2940	1970	2680	105	6120	Super large
HP038	Zhangzha	103.8795	33.2815	2100	2600	30	3024	Super large
HP039	Haizigou	103.7798	33.2572	2680	3210	320	2376	Super large
HP040	Jiangta	103.7229	33.1807	2900	3500	109	1512	Super large
HP041	Xiawujiao	104.2079	33.0401	1760	2050	272	1862	Super large
HP042	Yangshancun	104.2256	32.9932	1980	2160	300	552	Large
HP043	Miaozhoucun	104.1182	33.1106	2095	2350	159	880	Large
HP044	Xiewujiacun	104.1525	33.3561	2080	2620	205	3465	Super large
HP045	Yanmicun	104.1593	33.3387	1860	2390	287	720	Large
HP046	Dauluzhai	103.6787	33.5609	2500	2970	240	1485	Super large
HP047	Songpingcun	104.4738	33.2814	2160	2340	311	853.3	Large
HP048	Hetaoping	104.5753	32.9456	1380	1760	206	3060	Super large
HP049	Qiangqucun	104.4891	32.9252	1370	1750	108	1290.1	Super large
HP050	Zhentouba	104.4346	32.9078	1540	1890	161	1737.4	Super large
HP051	Huoyepo	104.7475	32.8533	970	1470	359	1224	Super large
HP052	Caopocun	104.3916	33.3423	1720	2090	123	276.5	Large
HP053	Xiacaodicun#1	104.3679	33.0141	1490	1740	92	320.8	Large
HP054	Xiacaodicun#2	104.3620	33.0098	1570	2000	121	792.1	Large
HP055	Congyacun#2	103.9428	33.2928	1980	2230	270	630	Large
HP056	Heihexiang#1	104.0022	33.5083	1940	2610	42	1536.8	Super large
HP057	Sandaocheng#1	103.9265	33.5436	2050	2750	36	3276	Super large
HP058	Heihetang#1	104.0595	33.3954	1750	2315	76	432	Large
HP059	Heihetang#2	104.0579	33.4029	1780	2135	84	305.5	Large
HP060	Qianghuotou	104.0656	33.4245	1885	2160	265	364	Large
HP061	Xinibashan	104.0472	33.4436	1845	2640	57	10400	Giant
HP062	Shimencun	103.9782	33.5940	2780	3270	194	833.7	Large
HP063	Banzangcun#1	103.8158	33.6059	2220	2660	34	1238.4	Super large
HP064	Banzangcun#2	103.8055	33.6077	2240	2735	346	1360	Super large
HP065	Duluyan	103.7647	33.6264	2640	2900	85	358	Super large
HP066	Tangnai	103.7403	33.5997	2440	2780	244	507	Large
HP067	Juguotaogao	103.7444	33.5917	2500	2600	244	225	Large
HP068	Kedemai	103.7507	33.5881	2550	2880	260	7177.5	Super large
HP069	Tongga	103.7052	33.6070	2530	2850	225	1540	Super large
HP070	Bduncun	103.6888	33.6131	2600	2960	221	2626.5	Super large
HP071	Zhimacun	103.6187	33.5884	2610	2970	200	1071	Super large
HP072	Yazhacun	103.9266	33.3173	2030	2300	183	326.7	Large
HP073	Congyayouzhan	103.9388	33.3053	1900	2295	282	330	Large
HP074	Longkangcun	103.9423	33.2864	1980	2230	299	979.2	Large

HP075	Pengbucun	103.8872	33.2712	2120	2790	80	270	Large
HP076	Zhangzhacun	103.8483	33.3071	2150	2440	191	9360	Super large
HP077	Borie	103.8183	33.2925	2355	2590	284	579.6	Large
HP078	Shabagouli#1	104.0004	33.2874	2570	2910	235	297	Large
HP079	Shabagouli#2	104.0065	33.2827	2860	2975	199	109.2	Large
HP080	Shabagouli#3	103.9993	33.2804	2740	3240	31	176.4	Large
HP081	Xuejiaba	104.0729	33.3520	1740	2150	209	1606.5	Super large
HP082	Anlezhaicun	104.2330	33.2981	1630	2175	308	1069.2	Super large
HP083	Shuanglongcun#1	104.2911	33.2536	1840	2145	247	367.2	Large
HP084	Shuanglongcun#2	104.2960	33.2661	2100	2420	285	522	Large
HP085	Shuanglongcun#3	104.2876	33.2608	2000	2440	158	276	Large
HP086	Shuanglongcun#4	104.2857	33.2531	1840	2145	106	302.4	Large
HP087	Xingshupo	104.3914	33.3027	1620	1980	87	4995	Super large
HP088	Zhulongnuocun	104.4231	33.4019	2000	2270	287	270.9	Large
HP089	Hawunuocun	104.4158	33.3720	2000	2175	258	112.2	Large
HP090	Dachengcun	104.3950	33.3087	1635	2065	248	1386.7	Super large
HP091	Xinhuacun	104.4065	33.2906	1740	2050	343	523.9	Large
HP092	Xinglongcun	104.4200	33.2250	1500	1780	270	562.5	Large
HP093	Dahaicun	104.4610	33.2432	1780	2145	340	480	Large
HP094	Wuxiacun	104.4519	33.1410	1520	1800	340	4384.8	Super large
HP095	Dafocun	104.4435	33.1092	1360	1600	290	301	Large
HP096	Mashanli	104.4631	33.1150	1540	1760	331	342.2	Large
HP097	Zhangjiagou	104.4775	33.1164	1760	1985	297	351	Large
HP098	Shuigouli	104.6745	33.1315	1570	2335	304	408	Large
HP099	Xiawujiaodui	104.1957	33.0455	1735	2380	61	398.4	Large
HP100	Shenglicun	104.1221	33.0551	1960	2300	153	690.2	Large
HP101	Punancun	104.2105	32.9742	2230	2560	138	8064	Super large
HP102	Gaoerfu#1	104.2158	33.2157	2150	2510	116	10500	Giant
HP103	Gaoerfu#2	104.2189	33.2281	2400	2600	170	4733.4	Super large
HP104	Zharu	103.9560	33.2518	2235	2650	225	903	Large
HP105	Nuoyede#1	103.9962	33.2120	2520	2700	212	248.4	Large
HP106	Nuoyede#2	104.0042	33.1959	2700	3200	118	633.6	Large
HP107	Panya	103.9009	33.2332	2390	2750	146	1458.6	Super large
HP108	Wucaichi	103.9327	33.0563	2910	3270	232	924	Large
HP109	Hekoucundui	104.0635	33.4160	1790	2130	287	120.7	Large
HP110	Hekoucun	104.0533	33.4185	1840	2200	52	218.4	Large
HP111	Ziyushan	104.0691	33.5131	2410	2930	226	2772	Super large
HP112	Gelishan	104.0791	33.5378	2510	3000	206	2418.4	Super large
HP113	Biershan	104.0465	33.5513	2500	2750	165	585.9	Large
HP114	Suanlishu	103.9396	33.4963	2370	2600	53	426.4	Large
HP115	Dongjiashan	103.9700	33.4969	2430	3075	257	2921.5	Super large
HP116	Yuwaqiao	103.8556	33.5942	2200	2610	331	630	Large
HP117	Wenjiagou	104.4620	33.0591	1244	1421	146	468	Large

HP118	Tangpugou#2	104.5264	33.0131	1062	1489	180	672	Large
HP119	Shaxucun	104.5112	33.0124	1054	1365	181	1224	Super large
HP120	Jiuguancunxia	104.5110	33.0037	1084	1504	337	685.4	Large
HP121	Gaojiashan	104.4954	33.0234	1064	1990	205	9880	Super large
HP122	Guojiapo	104.4775	33.0280	1087	1220	203	5760	Super large
HP123	Xinguancun	104.4589	33.0279	1127	1475	27	4110.6	Super large
HP124	Shijibaxiang#1	104.4525	33.0623	1126	1223	248	200.1	Large
HP125	Shijibaxiang#2	104.4517	33.0597	1150	1268	260	358.8	Large
HP126	Zhuyuanba#2	104.4260	33.0607	1115	1183	148	189.3	Large
HP127	Zhuyuanba#3	104.4202	33.0603	1136	1273	147	441	Large
HP128	Shuigouping	104.3658	33.0749	1190	1328	28	100.9	Large
HP129	Yinshan	104.6494	33.0638	1544	1755	211	330.5	Large
